# Xenon-mediated neuroprotection in response to sustained, low-level excitotoxic stress

**DOI:** 10.1038/cddiscovery.2016.18

**Published:** 2016-05-16

**Authors:** J Lavaur, M Lemaire, J Pype, D Le Nogue, E C Hirsch, P P Michel

**Affiliations:** 1 Institut National de la Santé et de la Recherche Médicale, U 1127, CNRS, Unité Mixte de Recherche (UMR) 7225, Sorbonne Universités, UPMC Univ Paris 06, UMR S 1127, Institut du Cerveau et de la Moelle épinière, ICM, Paris, France; 2 Air Liquide Santé International Medical R&D Paris, Saclay Research Center, Jouy-en Josas, France

## Abstract

Noble gases such as xenon and argon have been reported to provide neuroprotection against acute brain ischemic/anoxic injuries. Herein, we wished to evaluate the protective potential of these two gases under conditions relevant to the pathogenesis of chronic neurodegenerative disorders. For that, we established cultures of neurons typically affected in Alzheimer's disease (AD) pathology, that is, cortical neurons and basal forebrain cholinergic neurons and exposed them to L-trans-pyrrolidine-2,4-dicarboxylic acid (PDC) to generate sustained, low-level excitotoxic stress. Over a period of 4 days, PDC caused a progressive loss of cortical neurons which was prevented substantially when xenon replaced nitrogen in the cell culture atmosphere. Unlike xenon, argon remained inactive. Xenon acted downstream of the inhibitory and stimulatory effects elicited by PDC on glutamate uptake and efflux, respectively. Neuroprotection by xenon was mimicked by two noncompetitive antagonists of NMDA glutamate receptors, memantine and ketamine. Each of them potentiated xenon-mediated neuroprotection when used at concentrations providing suboptimal rescue to cortical neurons but most surprisingly, no rescue at all. The survival-promoting effects of xenon persisted when NMDA was used instead of PDC to trigger neuronal death, indicating that NMDA receptor antagonism was probably accountable for xenon’s effects. An excess of glycine failed to reverse xenon neuroprotection, thus excluding a competitive interaction of xenon with the glycine-binding site of NMDA receptors. Noticeably, antioxidants such as Trolox and N-acetylcysteine reduced PDC-induced neuronal death but xenon itself lacked free radical-scavenging activity. Cholinergic neurons were also rescued efficaciously by xenon in basal forebrain cultures. Unexpectedly, however, xenon stimulated cholinergic traits and promoted the morphological differentiation of cholinergic neurons in these cultures. Memantine reproduced some of these neurotrophic effects, albeit with less efficacy than xenon. In conclusion, we demonstrate for the first time that xenon may have a therapeutic potential in AD.

## Introduction

Noble gases such as xenon and argon have little propensity to participate in chemical reactions because of a filled valence shell. However, both xenon and argon possess interesting biological properties. Xenon is an approved anesthetic drug^1,2^ with organoprotective properties when administered alone^3,4^ or in combination with hypothermia.^5,6^ Xenon has been described as neuroprotectant in preclinical models of focal and global brain ischemia,^7–11^ spinal cord ischemia^12^ and traumatic brain injury.^13,14^ Some of the organoprotective and neuroprotective properties of xenon are also shared by argon.^14–16^ For example, argon can provide neuroprotection in acute brain slices subjected to oxygen and glucose deprivation,^16^ in rats subjected to intra-striatal injection of N-methyl-D-aspartate (NMDA)^16^ or to transient occlusion of the middle cerebral artery.^17^ Most recently, argon was also reported to reduce apoptosis of retinal ganglion cells after ischemia/reperfusion injury of the rat's eye.^18^

Activation of ATP-sensitive potassium channels or of two-pore potassium channels may explain some of the neuroprotective effects of xenon.^19,20^ Yet, it appears that xenon primarily acts by preventing NMDA receptor overexcitation under excitotoxic stress conditions.^7,14,21^ More specifically, xenon was reported to exert competitive inhibition at the glycine site of the NMDA receptor.^22^ Argon is protective too in experimental situations where neurodegenerative changes result from NMDA receptor overexcitation. However, argon distinguishes itself from xenon by not directly interfering with NMDA receptors and the putative targets of argon are still yet unknown.^15,23^

Excitotoxic stress mediated through NMDA receptors is most frequently associated to acute central nervous system insults such as ischemia and traumatic brain injury but chronic low-level overexcitation of these receptors by glutamate is also suspected to be one of the factors contributing to neuronal death in a number of chronic neurodegenerative conditions, including amyotrophic lateral sclerosis, Parkinson’s disease and Alzheimer’s disease (AD).^24,25^ The possible role of excitotoxic stress in AD is also suggested by reports showing that the two NMDA receptor antagonists, memantine and its close derivative nitromemantine provide some protection against AD progression in animal models of the disease.^26^ Memantine has also a small clinically detectable effect on cognitive dysfunction in AD patients.^27^

In the present study, we wished to assess the neuroprotective potential of xenon and argon in conditions that mimic chronic low-level excitotoxic stress as it may occur in AD. To this aim, we established cultures of neuronal populations most vulnerable in AD, that is, cortical neurons^28–30^ and basal forebrain cholinergic neurons.^29,31,32^ These cultures were submitted to mild excitotoxic stress by continued exposure to L-trans-pyrrolidine-2,4-dicarboxylic acid (PDC), a synthetic analog of L-glutamate that exerts inhibitory and stimulatory effects on glutamate uptake and efflux, respectively.^33–35^

We demonstrate for the first time that xenon can provide partial but sustained protection to cortical neurons undergoing neurodegeneration through mild excitotoxic stress. These protective effects were mimicked and amplified by two noncompetitive NMDA glutamate receptor antagonists, memantine and ketamine. Xenon also provided robust protection to PDC-treated cholinergic neurons in septal cultures but quite unexpectedly, it also exerted potent neurotrophic effects on these neurons.

## Results

### Xenon, but not argon, protects cortical neurons from death induced by chronic exposure to PDC

**Figure 1:**
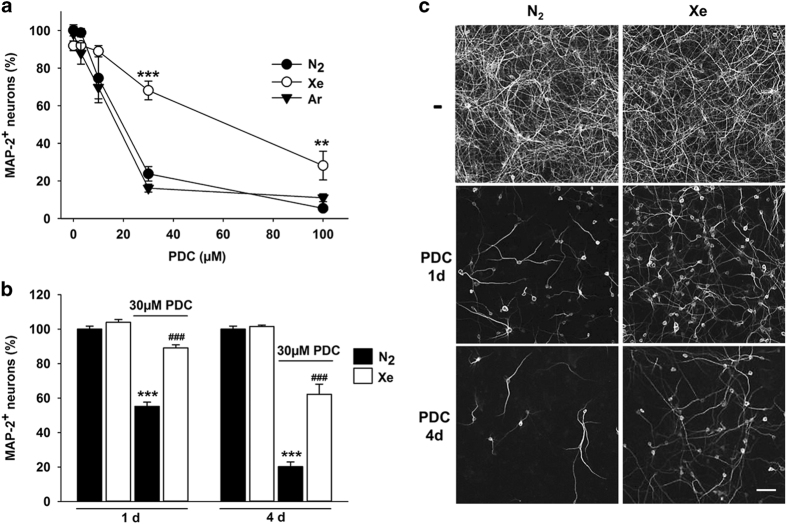
Xenon but not argon is protective against neuronal death induced by PDC in cortical cultures. (**a**) Survival rate of cortical neurons (MAP-2^+^ cells) in cultures exposed or not to PDC (3–100 *μ*M) for 4 days under an atmosphere containing 75% N_2_, 75% Xe or 75% Ar. Error bars indicate mean±S.E.M. (*n*=9). **P<0.01,***P<0.001 relative to cultures receiving a same concentration of PDC under N_2_ atmosphere. (**b**) Survival of cortical neurons exposed to 30 *μ*M PDC for 1 or 4 days in an atmosphere containing 75% N_2_ or 75% Xe. Error bars indicate mean ±S.E.M. (*n*=9). ***P<0.001 relative to control cultures maintained under N_2_ atmosphere and ^*###*^ *P*<0.001 relative to age-matched PDC-treated cultures under N_2_ atmosphere. (**c**) Fluorescence digitized images illustrating the neuroprotective effects provided by a gas atmosphere containing 75% Xe (*versus* 75% N_2_) in cortical cultures exposed for 1 or 4 days to 30 *μ*M of PDC. Scale bar 50 *μ*m.

Cortical cultures that had initially matured *in vitro* for 12 days were exposed to PDC, the synthetic analog of glutamate, to induce neurodegeneration. Cortical neurons identified by their content in microtubule-associated protein-2 (MAP-2) were affected when PDC was applied at concentrations equal or greater than 30 *μ*M for four consecutive days (Figure 1a). In a time course experiment carried out with 30 *μ*M PDC, we observed that neurodegeneration affected about 45% of cortical neurons after 1 day and more than 80% after 4 days of treatment (Figure 1b), indicating that neuronal demise developed progressively as a function of time in this experimental setting.

PDC-treated cortical neurons were largely protected from neurodegeneration when 75% nitrogen contained in the standard gas atmosphere was replaced by 75% xenon. After 1 and 4 days of treatment with 30 *μ*M PDC, the survival rate of cortical neurons in cultures chronically exposed to xenon was 89% (*versus* 55% in N_2_) and 62% (*versus* 20% in N_2_), respectively (Figures 1b and c). After 4 days of incubation with 100 *μ*M PDC, that is, treatment conditions where neuronal survival is below 5%, xenon remained neuroprotective even if its efficacy was reduced (Figure 1a). The replacement of nitrogen by argon failed to provide protection to cortical neurons undergoing chronic neurodegeneration regardless of the concentration of PDC used (Figure 1a). In the following experiments, we aimed to further characterize the neuroprotective effects of xenon.

### The effect of xenon is mimicked by pharmacological blockade of NMDA receptors

**Figure 2:**
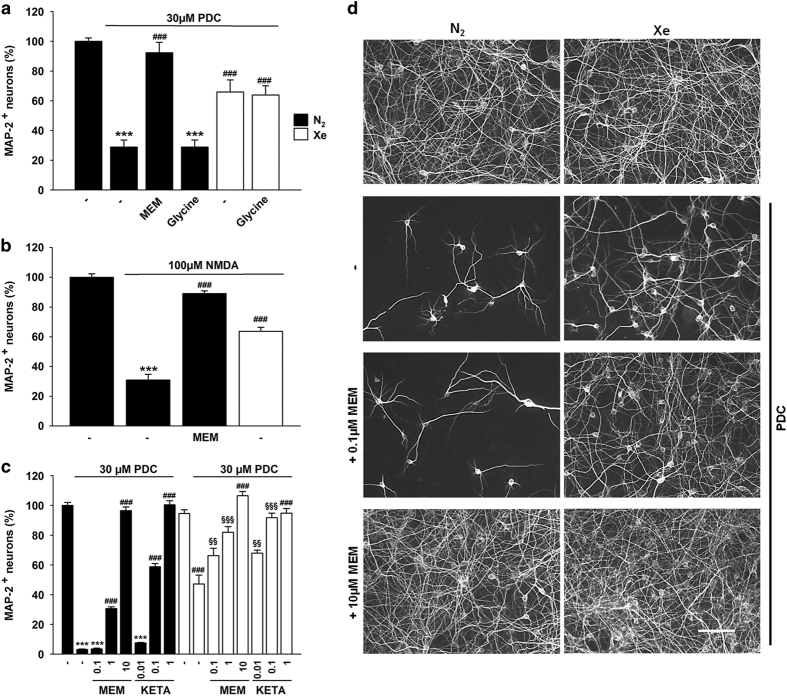
Role of NMDA glutamate receptors in xenon-mediated neuroprotection. (**a**) Survival rate of cortical neurons exposed to PDC (30 *μ*M) for 4 days and treated concurrently or not with the NMDA receptor blocker memantine (MEM, 10 *μ*M) or the amino acid glycine (1 mM) under a gas atmosphere containing 75% N_2_. Some cultures treated with PDC were also placed under a gas atmosphere containing 75% Xe and treated with or without glycine. Error bars indicate mean±S.E.M. *(n*=9). ***P<0.001 relative to control cultures under N_2_ atmosphere and ^*###*^ *P*<0.001 relative to PDC-treated cultures under N_2_ atmosphere. (**b**) Survival of cortical neurons exposed to NMDA (100 *μ*M) for 4 days under 75% N_2_ and the impact of a treatment with memantine (10 *μ*M) or 75% Xe. Error bars indicate mean±S.E.M. (*n*=9). ***P<0.001 relative to control cultures maintained under N_2_ atmosphere and ^*###*^ *P*<0.001 relative to PDC-treated cultures under N_2_ atmosphere. (**c**) Survival of cortical neurons exposed to PDC (30μM) for 4 days in the presence or not of various concentrations of memantine (0.1; 1; 10 *μ*M) or ketamine (0.01; 0.1; 1 *μ*M) under gas atmospheres containing either 75% N_2_ or 75% Xe. Error bars indicate mean±S.E.M. (*n*=9). ***P<0.001 relative to control cultures under N_2_ atmosphere, ^*###*^ *P*<0.001 relative to PDC-treated cultures under N_2_ atmosphere; ^§§^ *P*<0.01, ^§§§^ *P*<0.001 relative to PDC-treated cultures maintained under 75% Xe and PDC-treated cultures maintained under 75% N_2_ with a same concentration of memantine. (**d**) Fluorescence digitized images showing the impact that treatments with xenon alone or xenon+memantine (0.1; 10 *μ*M) exert on the survival of cortical cultures exposed to PDC. Scale bar 50 *μ*m.

To investigate the mechanisms underlying neuroprotection by xenon, we compared the effects of the noble gas to those of memantine, a noncompetitive antagonist of NMDA receptors. At an optimal concentration of 10 *μ*M, memantine protected about 90% of cortical neurons exposed for 4 days to 30 *μ*M PDC under a standard nitrogen atmosphere (Figure 2a), pointing to a key role of NMDA receptors in PDC-induced neurodegeneration. Xenon rescued 66% of cortical neurons in the same experimental setting. Glycine (1 mM), a co-agonist with glutamate at NMDA receptors did not intensify PDC-induced neuronal death under a control atmosphere and it also failed to reverse xenon-mediated neuroprotection (Figure 2a). When NMDA (100 *μ*M), the preferential agonist of NMDA receptors was used to trigger cortical cell death instead of PDC, xenon remained partially protective (Figure 2b). Memantine (10 *μ*M) rescued more than 89% of cortical neurons in the presence of 100 *μ*M NMDA. The efficacy of xenon to rescue cortical neurons against NMDA (100 *μ*M) or PDC (30 *μ*M) was roughly similar (Figure 2b).

### Xenon and NMDA receptor antagonists protect cortical neurons in a cooperative manner

Memantine and xenon are known to act as blockers of NMDA receptors.^26,36^ Therefore, we investigated whether the two molecules could cooperate to increase protection of cortical neurons (Figure 2c). Memantine at 0.1 *μ*M did not protect cortical neurons, but was able to substantially reinforce the action of xenon. The effect of xenon was also significantly improved with memantine at 1 *μ*M, a concentration that rescues in itself a substantial fraction of cortical neurons. No improvement of the effect of xenon was observed, however, when memantine was used at 10 *μ*M, that is, a concentration which already provides optimal neuroprotection *per se*. Of interest, another NMDA receptor antagonist ketamine also exerted cooperative protective effects with xenon (Figure 2c) when used at concentrations of 0.01 and 0.1 *μ*M that exert no and partial protective effects on their own, respectively. No cooperative effects were observed with a concentration of 1 *μ*M ketamine that was already optimally protective in itself.

### Impact of xenon on the uptake and release of glutamate

**Figure 3:**
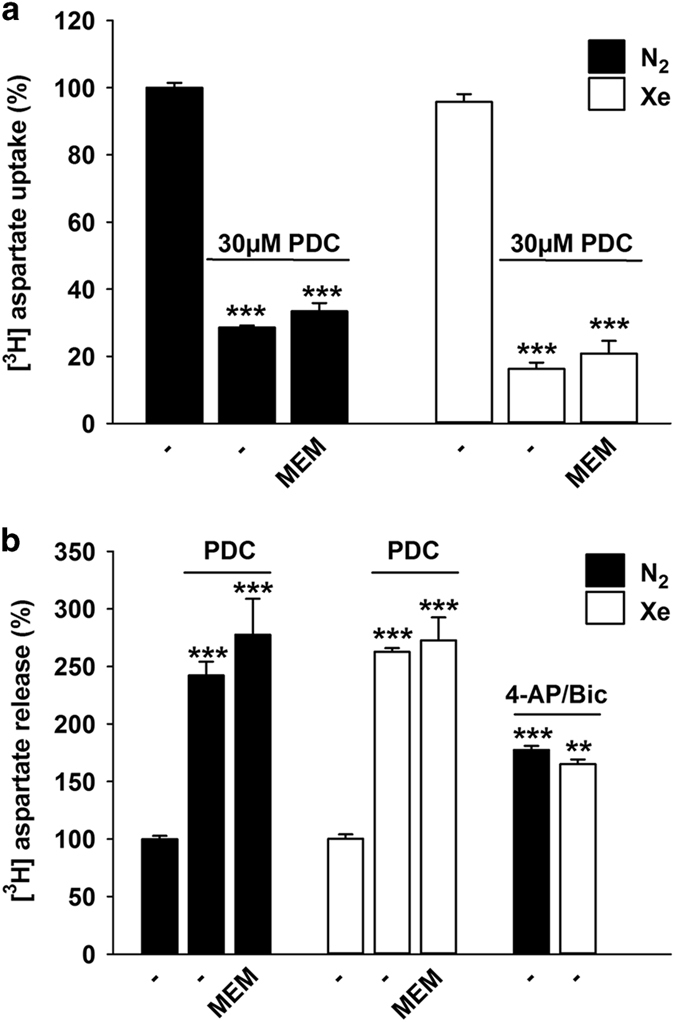
Impact of xenon on the uptake and release of [^3^H]-D-aspartate in cortical cultures exposed to PDC. (**a**) [^3^H]-D-aspartate uptake measured in cortical cultures exposed acutely or not to PDC (30 *μ*M) under an atmosphere containing 75% N_2_ or 75% Xe, in the presence or not of memantine (10 *μ*M). Error bars indicate mean±S.E.M. (*n*=6). ***P<0.001 relative to control cultures under N_2_ atmosphere. (**b**) [^3^H]-D-aspartate released in cortical cultures exposed or not to PDC (30 *μ*M) in an atmosphere containing 75% N_2_ or 75% Xe, in the presence or not of memantine (10 *μ*M). Error bars indicate mean±S.E.M. (*n*=9). ***P<0.001 relative to control cultures under N_2_ atmosphere. Impact of an atmosphere containing 75% Xe on [^3^H]-D-aspartate release evoked by a depolarizing treatment with 4-aminopyridine (4-AP; 2.5 mM) and bicuculline (Bic; 50 *μ*M). Error bars indicate mean±S.E.M. (*n*=9). **P<0.01, ***P<0.001 relative to control cultures maintained under N_2_ atmosphere.

We studied whether some of the effects of xenon resulted from direct interference with the mechanism of action of PDC. To do so, we used a non-metabolizable analog of L-glutamate, [^3^H]-D-aspartate, that labels the cytosolic and vesicular pools of endogenous excitatory amino acids.^37^ Our data show that an acute application of PDC (30 *μ*M) reduced the uptake of [^3^H]-D-aspartate by more than 70%. The uptake of [^3^H]-D-aspartate was not restored by xenon and it also remained impaired in the presence of memantine (10 *μ*M) (Figure 3a). Noticeably, the uptake of [^3^H]-D-aspartate was not significantly affected by xenon in control conditions.

Using cortical cultures initially pre-loaded with [^3^H]-D-aspartate, we found that PDC (30 *μ*M) stimulated the release of the tritiated neurotransmitter, which suggests that neuroprotection by xenon could potentially result from a reduction of PDC-evoked glutamate release. Xenon failed, however, to reduce the efflux of [^3^H]-D-aspartate upon PDC exposure (Figure 3b). Likewise, memantine did not interfere significantly with the release of [^3^H]-D-aspartate after PDC treatment.

We also assessed the impact of xenon on [^3^H]-D-aspartate release evoked by the K^+^ channel blocker 4-aminopyridine (2.5 mM) and the GABA_A_ receptor antagonist bicuculline (50 *μ*M), a pharmacological treatment known to physiologically activate neuronal glutamatergic synaptic activity.^38,39^ As expected, the depolarizing treatment enhanced [^3^H]-D-aspartate efflux but xenon failed to interfere with this process, which signifies that the noble gas had no significant impact on presynaptic glutamatergic release, in present conditions.

### Xenon prevents a death mechanism involving oxidative stress

**Figure 4:**
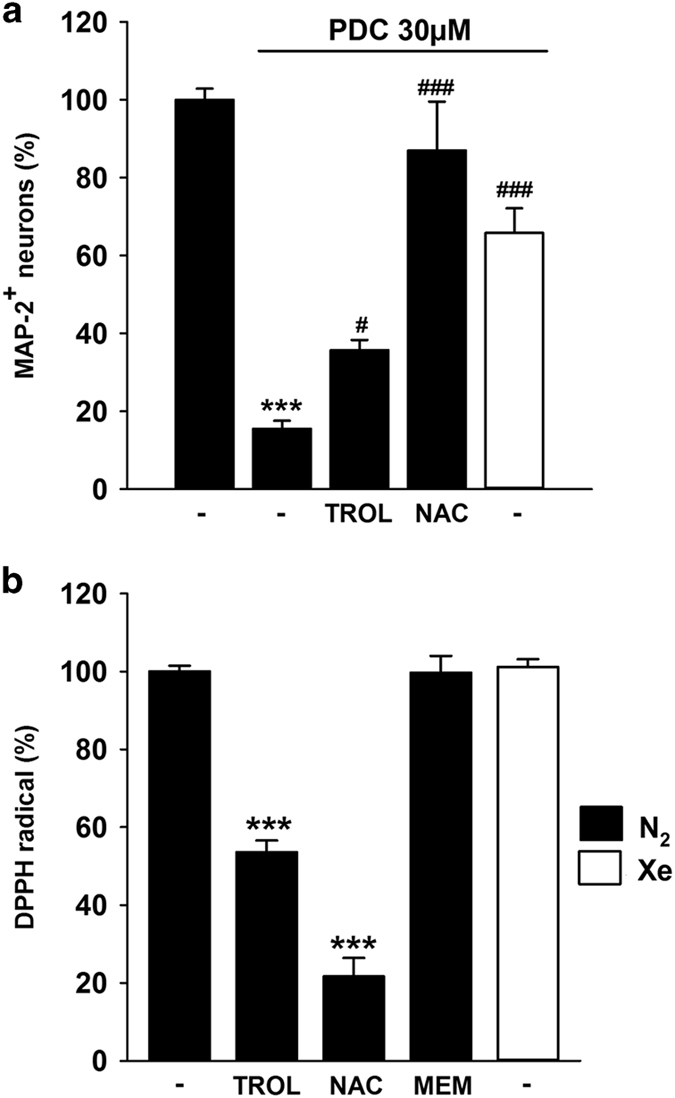
Xenon prevents a death mechanism that implicates oxidative stress. (**a**) Survival of cortical neurons in cultures exposed or not to PDC (30 *μ*M) for 4 days and treated or not concomitantly with Trolox (10 *μ*M) or NAC (30 *μ*M) under an atmosphere containing 75% N_2_. Comparison with cultures receiving the same concentration of PDC under an atmosphere containing 75% Xe. Error bars indicate mean±S.E.M. (*n*=6). ***P<0.001 relative to control cultures maintained under 75% N_2_ and ^*#*^ *P*<0.05, ^*###*^ *P*<0.001 relative to PDC-treated cultures maintained under 75% N_2_. (**b**) DPPH radical scavenging assay comparing the antioxidant potential of Trolox (10 *μ*M), NAC (30 *μ*M) and memantine (10 *μ*M) under 75% N_2_ to that of an atmosphere containing 75% xenon. Error bars indicate mean±S.E.M. (*n*=12). ***P<0.001 relative to controls maintained under 75% N_2_.

We established that oxidative stress was part of the death process inhibited by xenon. Indeed, the protection provided by 75% xenon against PDC (30 *μ*M) was reproduced partially by Trolox (10 *μ*M), a water soluble analog of vitamin E that operates as a scavenger of hydroxyl radicals^40^ and to a much larger extent by N-acetylcysteine (NAC) (30 *μ*M), a precursor of the intracellular antioxidant tripeptide glutathione (Figure 4a).^41^ To determine whether xenon had in itself the potential to operate as a free radical scavenger, we tested its activity using the 2-diphenyl-1-picrylhydrazyl (DPPH) cell-free assay.^42^ As expected, both Trolox (10 *μ*M) and NAC (30 *μ*M) reduced DPPH spontaneous oxidation. Their antioxidant capacity also matched their neuroprotective activity (Figures 4a and b). At variance, xenon did not demonstrate free radical scavenging activity (Figure 4b). Similarly, memantine (10 *μ*M) was totally ineffective in this assay.

### Xenon exerts both trophic and survival-promoting effects towards septal cholinergic neurons

**Figure 5:**
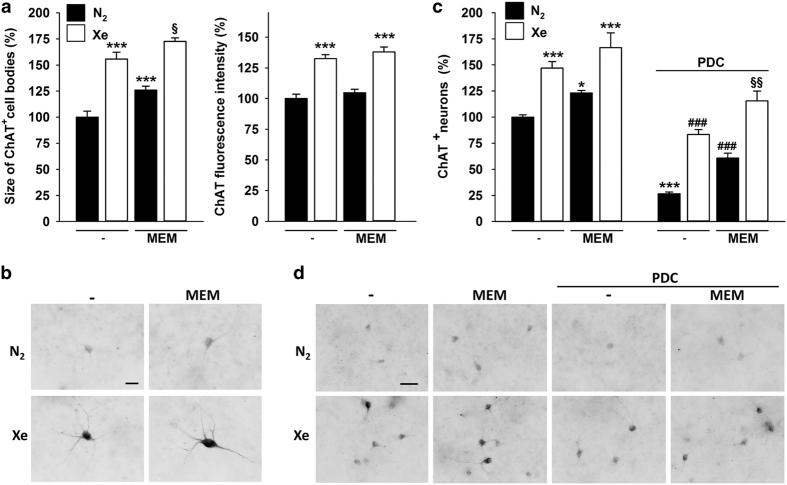
Xenon exerts both neurotrophic and neuroprotective effects for cholinergic neurons in septal cultures. (**a**) Left panel: Size of ChAT^+^ cell bodies in septal cultures exposed or not for 4 days to memantine (MEM; 10 *μ*M) under a gas atmosphere containing 75% N_2_ or 75% Xe. Right panel: ChAT fluorescence intensity in ChAT^+^ cell bodies exposed or not for 4 days to memantine (MEM; 10 *μ*M) under a gas atmosphere containing 75% N_2_ or 75% Xe. Error bars indicate mean±S.E.M. (*n*=25 randomly chosen neurons per condition). *** *P*<0.001 relative to control cultures maintained under 75% N_2_ and ^§^ *P*<0.05 relative to cultures maintained under 75% Xe and to cultures maintained under 75% N_2_ in the presence of memantine. (**b**) Fluorescence digitized images presented under an inverted format to illustrate the impact of the same treatments as in (**a**) onto cholinergic neurons. (**c**) Number of ChAT^+^ neurons in septal cultures maintained for 4 days under a gas atmosphere containing 75% N_2_ or 75% Xe and treated or not with PDC (30 *μ*M) in the presence or the absence of memantine (10 *μ*M). Error bars indicate mean±S.E.M. (*n*=18). * *P*<0.05, *** *P*<0.001 relative to control cultures maintained under 75% N_2_; ^*###*^ *P*<0.001 relative to PDC-treated cultures maintained under 75% N_2_ and ^§§^ *P*<0.01 relative to PDC-treated cultures maintained in 75% Xe and PDC-treated cultures maintained in 75% N_2_ in the presence of memantine. (**d**) Fluorescence digitized images showing the impact of the same treatments as in (**c**) onto cholinergic neurons. Scale bars: 25 *μ*m and 50 *μ*m in (**b**) and (**d**), respectively.

We also characterized the effects of xenon on septal cholinergic neurons, that is, a subpopulation of forebrain neurons particularly vulnerable to degeneration in the context of AD.^31,32^ Unexpectedly, cholinergic neurons identified by immunofluorescence detection of choline acetyltransferase (ChAT) exhibited much larger cell bodies when exposed transiently to 75% xenon between 12 and 16 days *in vitro* (DIV) (Figure 5a, left panel; Figure 5b). The intensity of ChAT immunolabelling was also significantly enhanced by the same gaseous treatment (Figure 5a, right panel; Figure 5b). Memantine (10 *μ*M) produced a smaller increase of the size of ChAT^+^ cell bodies than xenon and improved to some extent the effect that xenon exerted alone on this test parameter (Figure 5a, left panel), indicating that a combination of the two treatments may better preserve the morphology of cholinergic neurons. Memantine did not increase on its own the intensity of ChAT immunostaining in cholinergic cell bodies and did not improve the effect that xenon exerted on this test parameter (Figure 5a, right panel).

Of interest, there was also a substantial increase (46%) of neurons that were detectable by ChAT immunostaining in xenon-treated cultures in comparison with cultures exposed to a nitrogen atmosphere (Figure 5c). Memantine alone (10 μM) increased ChAT^+^ cell numbers in control cultures although to a lesser extent than xenon. The combination of the two treatments did not further increase cholinergic cell numbers in the absence of PDC (Figure 5c). When septal cultures were exposed to 30 *μ*M PDC between 12 and 16 DIV, only 26% of ChAT^+^ neurons survived compared with control cultures maintained under a standard nitrogen atmosphere. Under an atmosphere enriched with xenon, the survival rate of ChAT^+^ neurons was increased by more than threefold, reaching >80% of control values. The increase in ChAT^+^ neuron survival was less important with memantine (10 *μ*M). Interestingly, when xenon and memantine treatments were combined under PDC exposure, the number of cholinergic neurons exceeded that found in control cultures.

## Discussion

We demonstrate for the first time that the noble gas xenon can provide partial but sustained protection to AD vulnerable neurons (i.e., cortical neurons and septal cholinergic neurons) that undergo degeneration though chronic low-level excitotoxic stress. The protective effect of xenon was not reproduced with the other noble gas argon. We established that xenon interferes with a death pathway in which NMDA receptors and reactive oxygen species intervene crucially. Interestingly, xenon exerted cooperative neuroprotection with two noncompetitive NMDA receptor channel antagonists, memantine and ketamine. When evaluating the impact of xenon in septal cultures, we found that the noble gas was not only robustly neuroprotective for cholinergic neurons but that it also exerted potent trophic effects on them.

### Xenon operates as a neuroprotectant against low-level excitotoxic stress

Previous studies have shown that xenon is neuroprotective in cellular or animal models that mimic acute brain ischemic or traumatic insults.^7–11,43^ The present work explored the neuroprotective potential of xenon in two model systems that mimic degenerative changes linked to the AD pathology.

In addition to histopathological hallmarks such as extracellular deposition of amyloid plaques and intracellular accumulation of neurofibrillary tangles, AD is also characterized by a progressive loss of neurons in several brain areas, in particular the cerebral cortex.^28,30,44^ We therefore established a model system of cortical cultures in which neuronal cell death was triggered by continued application of a synthetic glutamate analog PDC in order to generate slowly evolving excitotoxic stress, a likely component of neuronal death in the AD pathology.^24,45,46^ As expected, excitotoxic cell death induced by PDC progressed relatively slowly in this experimental setting, presumably because neurodegenerative changes are caused by the progressive accumulation of endogenous glutamate in the extracellular milieu.^33–35^

When the conventional cell culture atmosphere was modified with a gas mixture, including the same amount of oxygen (20%) and carbon dioxide (5%) but 75% xenon instead of nitrogen, PDC-induced cortical cell death was reduced to a significant extent, 1 and 4 days after the onset of neurodegeneration. This observation is consistent with previous studies reporting on the neuroprotective effects of xenon in paradigms that mimic acute excitotoxic insults.^7–11,43^ However, to our knowledge, this is the first time that the neuroprotective potential of xenon is established in an experimental setting where neurodegenerative changes are progressively evolving. Incidentally, our data also show that control cortical neurons perfectly tolerate a long-term exposure to xenon which is coherent with brain histological studies carried out in newborn pigs inhaling the noble gas during extended periods of time.^47^

### Xenon does not interfere with PDC effects on glutamate uptake or release

In agreement with previous reports,^33–35^ we found that PDC reduced the reuptake of glutamate and stimulated its release in the extracellular milieu. Hence, we hypothesized that neuroprotection by xenon against PDC could result from a reduced build-up of glutamate in the culture medium in response to the gaseous treatment. The effects of PDC on the uptake and release of glutamate were, however, not significantly impacted by xenon. The synaptic release of glutamate, which can be mimicked physiologically by a combined treatment with the K^+^ channel blocker 4-aminopyridine and the GABA_A_ receptor antagonist bicuculline,^38,39^ also remained unaffected under xenon exposure. This set of data clearly indicates that xenon is unlikely to afford protection by interfering with a presynaptic mechanism linked to the uptake or release of glutamate.

### Xenon reduces NMDA receptor-mediated neurodegeneration

Memantine and ketamine, both of which operate as noncompetitive glutamate NMDA receptor antagonists, were able to mimic the protective effect of xenon against PDC, indicating that xenon was possibly acting itself by antagonism of this receptor subtype. Xenon is well known to reduce the activation of NMDA receptors,^48^ possibly by interfering at the binding site for glycine,^14,21^ a co-agonist for NMDA receptor activation, which modulates the amplitude and time course of glutamate-elicited responses.^49,50^ Xenon-mediated neuroprotection remained, however, unaffected when the culture medium used to maintain the cultures, that is, Neurobasal medium, was supplemented with an excess of glycine. This is rather unexpected because glycine has been reported previously to reverse neuroprotection by xenon in an *in vitro* model of traumatic brain injury.^22^ This result is, however, explainable considering the fact that glycine is present intrinsically in Neurobasal culture medium at a concentration of 400 *μ*M, which is saturating for the NMDA receptor glycine-binding site.^22^ Even at saturating concentrations of glycine, xenon conserved the capacity to reduce NMDA currents, significantly.^22^ This suggests that NMDA receptors may still represent the neuroprotective target of xenon in the present paradigm but via a mechanism that is not sensitive to glycine and remains to be elucidated. Still consistent with the view that xenon operated via an antagonistic effect on NMDA receptors, the noble gas remained strongly protective when NMDA, the prototypical agonist of NMDA receptors was used instead of PDC to induce cortical cell death.

### Unlike xenon, argon is not neuroprotective under chronic low-level excitotoxic conditions

Similar to xenon, argon has been reported to have strong organoprotective and neuroprotective effects.^16,17^ Neuroprotective effects have been described in a large variety of culture and animal models of acute excitotoxic stress.^15^ Therefore, the lack of protection of argon in the present study was rather unexpected. Unlike xenon, argon does not interfere with NMDA receptors.^14,15^ In fact, argon is believed to directly activate intracellular pathways that control the expression of anti-apoptotic proteins such as BCL-2.^51^ This may be one of the reason why argon is not neuroprotective in a context of chronic neurodegeneration.

### Xenon provides cooperative rescue with memantine or ketamine

Like xenon, the two noncompetitive NMDA antagonists, memantine and ketamine were protective in the present paradigm. This led us to evaluate the efficacy of treatments combining xenon with each of these two molecules. Most interestingly, we found that low concentrations of memantine and ketamine that afford no or partial protection on their own against PDC, had the capacity to markedly improve the rescue of cortical neurons provided by xenon, alone. This cooperative effect may be because of the fact that xenon on one hand, and memantine and ketamine on the other hand, operate via distinct mechanisms. Indeed, memantine and ketamine possess a blocking site situated within the NMDA channel pore,^49,52^ whereas xenon operates most likely elsewhere on the receptor.^22^ Cooperative effects of this kind may be of potential clinical interest, in particular in the case of memantine, which is an approved drug for the treatment of AD.^27^

### Xenon prevents a death process involving oxidative stress

Oxidative stress participates actively in NMDA receptor-mediated neurodegeneration,^53^ which explains why pharmacological or genetic manipulations that restrain the production of reactive oxygen species have been reported to partially block neuronal death in acute models of excitototoxic stress.^54,55^ Here, we established that Trolox, a scavenger of hydroxyl radicals,^40^ and to a larger extent, NAC, a synthetic precursor of the intracellular antioxidant tripeptide glutathione,^41^ provided protection to cortical neurons in cultures chronically exposed to PDC. A number of gases including hydrogen, hydrogen sulfide and carbon monoxide,^56–58^ have been reported to possess antioxidant properties but experimental evidence is lacking in the case of xenon. The use of the cell-free DPPH assay revealed that xenon does not behave as an antioxidant in comparison with Trolox and NAC used as reference molecules. Therefore, one may assume that xenon prevented the propagation of oxidative stress-mediated damage, indirectly, through its capacity to limit NMDA receptor overstimulation.

### Xenon exerts both neuroprotective and neurotrophic effects for septal cholinergic neurons

We were also interested in evaluating the impact of xenon onto basal forebrain septal cholinergic neurons, that is, a population of neurons that is also particularly vulnerable to degeneration in the context of the AD pathology.^29,31,32^ Xenon was not only robustly protective for cultured septal cholinergic neurons exposed to PDC but unexpectedly the gaseous treatment also had the capacity to strongly promote cholinergic neuron maturation in the absence of PDC. Most strikingly, xenon enhanced the size of cholinergic cell bodies and stimulated the cellular expression of the cholinergic marker protein ChAT in neurons immunopositive for this protein. The cholinotrophic effect of xenon extended presumably to a subset of dormant neurons expressing undetectable levels of the ChAT enzyme before initiation of the gaseous treatment. Indeed, the noble gas increased cholinergic cell numbers (i.e., ChAT^+^ cells) by more than 40% in the absence of PDC. Even with a lower efficacy, memantine reproduced some of the cholinotrophic effects of xenon, indicating that NMDA receptor antagonism probably played a role in these effects. Present observations appear coherent with previous studies showing that NMDA receptor blockade can promote expression of cholinergic traits during development in subsets of forebrain glutamatergic neurons both *in vitro* and *in vivo*.^59,60^

Quite remarkably, the trophic effects of xenon were observed despite the supplementation of the culture medium with nerve growth factor (NGF), a neuropeptide with cholinotrophic properties.^61^ This is not so surprising considering the fact that the NGF regimen used in this study (3×50 ng/ml over a period of 16 days) failed to promote full maturation of cholinergic neurons. This signifies that present culture conditions may be particularly favorable to reveal the cholinotrophic properties of xenon. The neurotrophic effects of xenon for cholinergic neurons are of interest as there is evidence from experimental lesions in animals and post-mortem human studies that these neurons are depleted of phenotypic markers well before dying in AD.^62,63^ Whether induction or restoration of cholinergic traits can also occur with xenon in a pathological context remains, however, to be established.

As mentioned previously, xenon also provided robust protection to cholinergic neurons exposed to PDC. The noble gas was proportionally more efficacious to rescue cholinergic neurons than the bulk population of neurons that undergoes neurodegeneration in septal cultures (not shown) or in cortical cultures. This suggests that the trophic effects of xenon also occurred in PDC-treated septal cultures despite ongoing neurodegenerative changes. Coherent with this view, memantine, which was less effective than xenon in increasing the number of ChAT^+^ neurons in control cultures, was also comparatively less efficacious than the noble gas to protect cholinergic neurons against PDC exposure.

Overall, our data demonstrate that the noble gas xenon exerts robust protective effects for cortical neurons and subcortical cholinergic neurons that are preferentially vulnerable in AD. Besides exerting true neuroprotective effects, xenon can also operate as a potent trophic factor for cholinergic neurons. Although any extrapolation from our *in vitro* work to a pathologic situation must be regarded as speculative, our data raise the intriguing possibility that xenon may have the potential to halt neurodegenerative events associated with the progression of the AD pathology.

## Materials and Methods

### Pharmacological reagents

The non-metabolizable analog of L-glutamate PDC, the preferential agonist of NMDA receptors NMDA and the NMDA receptor antagonist memantine, were all purchased from Tocris Biotechne (Lille, France). Ketamine was obtained from Virbac (Carros, France). The GABA_A_ receptor antagonist bicuculline, the non-selective K^+^ channel blocker 4-aminopyridine, NGF 2.5S, the two antioxidant molecules NAC, Trolox and the DPPH free radical were from Sigma–Aldrich (Saint-Quentin Fallavier, France).

### Cortical and septal neuronal cultures

Animals were housed, handled and taken care of in accordance with recommendations of the Guide for the Care and Use of Laboratory Animals of the National Institutes of Health (NIH Publication no. 85-23, revised 1996) and the European Union Council Directives (86/609/CEE). Experimental procedures were authorized by the ethical committee on animal experiments Charles Darwin n°5.

Cortical cultures were prepared using gestational age 15.5 embryos harvested from Wistar female rats (Janvier LABS, Le Genest St Isle, France), which had been previously killed with a sodium pentobarbital overdose followed by cervical dislocation. Upon isolation, brain cortices were dissociated by mechanical dissociation in Leibovitz L15 culture medium (Sigma–Aldrich) according to the protocols previously described for mesencephalic cultures.^64^ Dissociated cells in suspension were then seeded at a density of 0.3–0.4×10^5^ cells/cm^2^ onto Nunc 48-well multi-dish plates (Thermo Fisher Scientific, Roskilde, Denmark) pre-coated with 1 mg/ml polyethylenimine diluted in borate buffer, pH 8.3. Cultured cells were maintained for maturation in Neurobasal medium (Gibco, Saint Aubin, France) supplemented with a B27 cocktail minus antioxidants (Gibco), a N2 mix, 2 mM glutamine and 100 IU/ml penicillin/streptomycin.^34^ Glial cell proliferation was halted by adding 0.5 *μ*M of the antimitotic cytosine arabinoside, not later than 12 h after plating.

Septal cultures were obtained by dissection of the forebrain area that contains cholinergic neurons and gives rise to the medial septal nucleus and the nucleus of the diagonal band of Brocca.^61,66^ After trituration of brain tissue pieces, septal cells in suspension were plated at a density of 1–1.2×10^5^ cells/cm^2^. Septal cultures were maintained using the same conditions as those described for cortical cultures, except that NGF (50 ng/ml) was added to the cultures after plating, and then at DIV 7 and 12 to stimulate the phenotypic differentiation of cholinergic neurons.^61,65^ Cortical and septal cultures were maintained up to the indicated times before being processed for assessment of test parameters.

### Culture exposure to gases

After pharmacological treatments, 48-well multi-dish plates containing cultured cells were disposed above a metallic base plate receiving atop of it a Plexiglas incubation chamber. The two pieces were then screwed together to produce an air-tight seal. Humidification of the chamber was achieved by disposing a multi-dish plate wherein culture wells filled with distilled water were placed directly in contact with the inside atmosphere. When needed, the pre-defined gas mixture comprising 20% O_2_, 5% CO_2_ and 75% of the test gas was injected at a flow rate of ~10 l/min into the incubation chamber with the inlet and outlet valves open. When the CO_2_ concentration measured at the outlet with a gas analyzer reached 5%, the injection was stopped and the chamber was sealed by turning off the inlet and outlet valves. The incubation chamber was then placed in a conventional incubator at 37 °C for incubation times indicated in the manuscript. We generally used pre-defined gas mixtures provided by Air Liquide (France). In some experiments, controlled gas atmospheres were also delivered to incubation chambers using a gas mixing system (GasMix, Alytech, Juvisy/Orge, France) supplied by tanks filled by individual pure gases (Air Liquide).

### Protein detection by immunofluorescence

The cultures, fixed with 4% formaldehyde in Dulbecco’s phosphate-buffered saline (PBS), were washed twice with PBS before an incubation step at 4 °C for 48–96 h with primary antibodies diluted in PBS containing 0.2% Triton X-100 to improve cell membrane permeability. A monoclonal MAP-2 antibody (clone AP-20, #M2320; Sigma–Aldrich) diluted 1/200 was used as the pan-neuronal marker and a goat anti-ChAT affinity purified antibody diluted 1/100 (#AB144P; Merck Millipore, Darmstadt, Germany) as the cholinergic marker. Detection of primary antibodies was performed with an Alexa Fluor-555 conjugate of an anti-mouse IgG antibody (1/500) or an Alexa Fluor-555 conjugate of an anti-goat antibody (1/1000) both obtained from Life Technologies (Saint Aubin, France).

### Assessment of neuronal viability

The survival of all neurons regardless of their neurotransmitter phenotype was monitored by counting MAP-2^+^ cells. Briefly, fluorescent images of cultured cells were acquired with an Eclipse TE-2000 inverted fluorescent microscope (Nikon, Champigny-sur-Marne, France) equipped with an ORCA-ER digital camera (Hamamatsu Photonics, Massy, France) operated with the HCI software (Hamamatsu Corp., Bridgewater, NJ, USA). In each culture well, 10 digitized images randomly acquired with a ×10 objective were taken for cell counting using the image processing program Image J. Counting of ChAT^+^ neurons was performed on digitized images acquired with a ×10 objective mounted onto an Arrayscan XTi automated workstation equipped with the HCS Studio Software (Thermofisher Scientific, Courtaboeuf, France). Mosaic reconstruction of partially overlapping digitized images allowed the counting of ChAT^+^ neurons over >60% of the surface area of each culture well.

### Markers of cholinergic neuron maturation

To estimate the impact that test treatments could have on the maturation of cholinergic neurons, digitized images of ChAT^+^ neurons were taken randomly with the Nikon Eclipse TE-2000 microscope using a ×40 objective and identical acquisition parameters. Images were used to quantify the surface area and the fluorescence intensity of positive cell bodies using the MCID analysis software (InterFocus Imaging Ltd, Cambridge, UK). Specific ChAT fluorescence intensity was corrected by subtracting background intensity levels for each treatment condition. All results were expressed in percent of control values.

### [^3^H]-D-aspartate uptake and efflux assays

[^3^H]-D-aspartate, a non-metabolizable analog of L-glutamate that labels the cytosolic and vesicular pools of endogenous excitatory amino acids,^33,66^ was used to estimate the uptake and release of glutamate. Briefly, the culture medium was removed and the cultures were allowed to recover for 5 min at 37 °C in PBS supplemented with 5 mM glucose before further processing. The uptake was initiated by the addition of 1 *μ*l of [2,3-^3^H]-D-aspartate (13 Ci/mmol; Perkin Elmer, Waltham, MA, USA) to culture wells exposed or not to different test treatments in the presence of gas atmospheres enriched or not with xenon. After 30 min, the cultures were washed twice with cold PBS to remove the tritiated amino acid in excess and the radioactivity accumulated intracellularly was recovered by scrapping off cells in distilled water. Samples were then assessed by liquid scintillation spectrometry. The release of [2,3-^3^H]-D-aspartate was carried out in cultures that were pre-loaded for 30 min with the tritiated amino acid, washed twice with PBS and then exposed to various test treatments in the presence of gas atmospheres enriched or not with xenon. The fraction of radioactivity recovered in the incubation medium during the next 30 min was collected for assessment by liquid scintillation spectrometry.

### Free radical-scavenging assay

Radical-scavenging activities were measured using DPPH, a hydrazyl derivative producing a stable free radical with a deep purple color when diluted in pure ethanol.^41^ Briefly, 225 *μ*l of a DPPH ethanol solution (0.025 mg/ml) were distributed into individual wells of a Nunc 96-well microplate. The volume was then completed to 250 *μ*l by addition of the test compounds diluted in ethanol or by ethanol, alone. Blanks contained 250 *μ*l of pure ethanol instead of DPPH. The microplate shaken vigorously during 10 s was then allowed to stand for 1 h in the dark at room temperature under atmospheres containing either 75% nitrogen or 75% xenon. This was followed by measurement of the absorbance at 517 nm using a Spectramax M4 multiplate reader (Molecular Devices, Sunnyvale, CA, USA). Blank values obtained by replacing DPPH with pure ethanol were subtracted from test samples.

### Statistical analyses

Data expressed as means±S.E.M were analyzed using the SigmaPlot 12.5 software (Systat Software Inc, San Jose, CA, USA). Multiple comparisons against a single reference group were made by one-way ANOVA with Dunnett’s test *post hoc* analysis. When all pairwise comparisons were required, one-way ANOVA was followed by the Student-Newman-Keuls *post hoc* test.

## Abbreviations

AD: Alzheimer’s disease
ChAT: Choline acetyltransferase
DIV: day *in vitro*
DPPH: 2-diphenyl-1-picrylhydrazyl
MAP-2: microtubule-associated protein
NAC: N-acetylcysteine
NGF: Nerve growth factor
NMDA: N-methyl-D-aspartate
PBS: Dulbecco’s phosphate-buffered saline
PDC: L-trans-pyrrolidine-2,4-dicarboxylic acid
